# Application of Electromagnetic Braking to Minimize a Surface Wave in a Continuous Caster

**DOI:** 10.3390/ma16031042

**Published:** 2023-01-24

**Authors:** Saswot Thapa, Mingqian Wang, Armin K. Silaen, Mauro E. Ferreira, Wesley Rollings, Chenn Zhou

**Affiliations:** 1 Center for Innovation through Visualization and Simulation (CIVS) and Steel Manufacturing Simulation and Visualization Consortium (SMSVC), Purdue University Northwest, Hammond, IN 46323, USA; 2 Nucor Steel Decatur, LLC, 4301 Iverson Blvd, Trinity, AL 35673, USA

**Keywords:** continuous casting, electromagnetic braking, magnetohydrodynamics, surface standing wave, primary cooling, Lorentz force

## Abstract

The turbulent flow in the mold region drastically influences the quality of steel produced during continuous casting. The flow itself can lead to surface defects or slag entrainment based on the formation. A high surface wave can lead to fluctuations and the instability compromises the quality of the steel produced, as well as entrain the slag. To regulate the flow, electromagnetic forces can be applied in the mold, dampening the local turbulent flow. As the electrically conductive molten steel interacts with the induced magnetic field, it reduces the velocity of the steel jet released from the ports of the submerged entry nozzle. Utilizing Star-CCM+, a simulation-based study is conducted modeling the impact of Electromagnetic braking (EMBr) on the flow formation and surface standing wave. Specifically, a parametric study is conducted investigating the impact of submergence entry nozzle (SEN) depth and mold width with applied EMBr. Per the simulation-based study conducted increasing the EMBr strength from 2975 G to 4350 G reduced the average surface wave height by 12.5% and volume of flux rate of decrease by 4.25%. Additionally, increasing the SEN depth from 110 mm to 350 mm increased the average wave height by 19% and volume of flux rate of decrease by 2.6%. Lastly, increasing the mold width from 1.067 m to 1.50 m increased average wave height by 8.71% and volume of flux rate of decrease by 0.9%.

## 1. Introduction

Continuous casting (CC) is a widely used process to solidify molten steel. The process begins with the tundish where molten steel is poured from the ladle. From the tundish, the molten steel is ferro-statically pushed into the upper tundish nozzle (UTN) and then into the submerged entry nozzle (SEN). The SEN regulates the flow of molten steel injected into the mold and the formation of the solidified shell. The shell is then pulled from primary cooling into secondary cooling by rollers where further cooling occurs utilizing spray nozzles. Once the molten steel fully solidifies it can be cut by a flame torch or a shear to create a bloom, billet, or slab, which can be processed to form steel products. Primary cooling is a critical process of CC, and it is the last place where steel quality can be optimized. Maintaining stable flow near the top surface of the mold helps produce higher quality steel. One way this can be achieved is by maintaining the surface defects by regulating the flow with electromagnetic braking (EMBr). By reducing the molten flow with magnetic flux, the surface wave fluctuation can be reduced as well [1].

During the continuous casting of the steel, the quality of cast products is predominantly affected by the flow dynamics at the meniscus level of the mold [1]. Abrupt mold surface velocity variation brings asymmetry to the surface directed flow and thus can lead to occurrences of instability between the molten steel and slag layer interface. As a result of the surface wave instability, the slag layer can be entrained into the molten pool to form internal and surface defects in the final cast products [2]. It is noted that intermittent defects occur due to excessive transient fluctuations at the liquid level [3]. The slag entrainment and surface defects from wave fluctuations will occur if the surface flow is too fast or if the wave profile is not flat enough [3,4].

In contrast, irregular sluggish surface flow could generate low and non-uniform surface temperatures. This induces inadequate meniscus freezing, slag melting and infiltration, hook formation, and surface defects [2]. The local turbulent flow can be dampened instantaneously with the electromagnetic forces generated by EMBr. As a result of the interaction between the electrically conductive molten steel and the induced magnetic field, a Lorentz force is generated acting against the molten steel [5]. The Lorentz force dampens the molten steel, ultimately slowing the velocity of the steel jet released from the ports of the SEN and lowering the surface wave [5]. An appropriate EMBr strength can reduce the surface standing wave, which provides a calmer and hotter meniscus, thus providing uniform solidification. If the EMBr strength is too high, the Lorentz force acting against the molten steel can be too strong, leading to meniscus freezing, while lower-strength or no EMBr can cause large surface fluctuations resulting in uneven solidification, vertices, impingement zones, and surface defects [6].

In general, there are three types of EMBr unit used in industrial practice, namely the local type, the ruler type, and the multi-type. Li et al. discussed the braking effect per magnetic field arrangement and showed the advantages and disadvantages for each type of EMBr unit. For the local type EMBr, it can be further divided into four main types: the first generation local type EMBr, upper jet-pattern EMBr, jet-pattern EMBr, and local vertical EMBr [7]. The first generation local type EMBr was used and simulated in our research.

In 2007, Thomas et al. developed a mathematical model simulating flow control with EMBr during CC. The validated model utilized a coupled magnetohydrodynamics (MHD) model with a single-phase k-epsilon (k-ε) turbulence model. To validate the model, the simulation results were compared with experimental measurements of the surface wave captured through oscillation marks on the slab surface. A gauss meter was utilized to measure the EMBr fields in the mold cavity for the model with the caster design provided by Nucor Steel Decatur. From the conducted computational analysis, it was found that increasing the EMBr strength at a constant SEN caused deeper jet penetration, weaker upper recirculation, weaker meniscus velocity, and weaker surface wave. Increasing the SEN depth at a constant EMBr strength resulted in deeper jet penetration, larger meniscus velocity, and larger surface wave [8]. It is crucial that surface wave be minimized as excessive surface velocity and surface wave can entrain the mold slag inclusions leading to surface defects. This model can further be improved incorporating the slag and air layer with surface tension defined between the layers to account for the dynamics during casting.

In 2017, Jin et al. published a manuscript focusing on the effect of the EMBr and SEN submergence depth on a turbulent flow during casting. This paper also states that because of the characteristics of a turbulent flow in the mold, the creation of surface defects and slag entrainment is caused due to a larger surface wave. Unlike the static local EMBr applied to the broad face of the mold utilized in the study presented in this manuscript, Jin’s work utilizes a double-ruler EMBr configuration. The purpose of this configuration is that it is strategically placed in the mold to slow down or speed up surface velocity to stabilize meniscus stability. The model utilized in this study is a coupled turbulent MHD with solidification. Per the cooling data utilized in the mold, the shell thickness is extracted and used as a domain for the molten steel flow for the MHD model. Per the conducted study, applied EMBr caused the flow in the nozzle to be more uniform, increasing the downward velocity and momentum along the SEN walls. Lastly, the applied EMBr reduced the surface velocity by 0.05 m/s, as well as reducing the surface fluctuations [9].

As for the impact of EMBr on steel solidification, Wang et al. studied the superheat transport and distribution in the mold. The results showed that the EMBr will move up the superheat zone and increase the meniscus temperature, which can help the melting of the slag and reduce the surface defects [10]. Microscopically, EMBr also has an effect on grain growth. Xu et al. studied the effects of electromagnetic stirring (EMS) on dendrite growth and segregation. The results show that EMS can promote columnar-equiaxed transition and reduce the segregation [11].

In this research, a series of numerical simulations considering the application of EMBr based on realistic caster and casting conditions was conducted, and the effects of EMBr application on surface standing wave and flux entrainment were focused on.

## 2. Methodology

### 2.1. Governing Equations

The flow is modeled utilizing Navier−Stokes equations based on the model developed by Chaudhary et al. [12]. The following are the governing equations for mass and momentum conservation. The model utilized is isothermal and does not consider heat transfer.

(1) $$\frac{\partial }{\partial t}\left(\int_{V}\rho dV\right)+\oint_{A}\rho \vec{v}\cdot d\vec{a}=0\;$$

(2) $$\frac{\partial u_{i}}{\partial t}+\frac{\partial u_{i}u_{j}}{\partial x_{j}}=-\frac{1}{\rho }\frac{\partial \rho^{*}}{\partial x_{i}}+\frac{\partial }{\partial x_{j}}\left(\left(v+v_{s}\right)\left(\frac{\partial u_{i}}{\partial x_{j}}+\frac{\partial u_{j}}{\partial x_{i}}\right)\right)+\frac{1}{\rho }{\vec{F}}_{L}$$

In Equation (1), for conservation of mass, ρ is density, A is area, and v is velocity. In Equation (2), for conservation of momentum, p is pressure, u is the velocity component, *p* is pressure, $\rho^{*}$ is static pressure, v is viscosity, $v_{s}$ is sub grid viscosity, and $F_{L}$ is the Lorentz force.

### 2.2. The Magneto-Hydrodynamics Model

To model the interaction between the molten steel and the induced magnetic field, a coupled magneto-hydrodynamics (MHD) model is integrated into the turbulent fluid flow simulation [13]. The model is theorized as shown in Equation (3), where ${\vec{J}}_{L}$ is the total electric current density, σ is the electrical conductivity, and $\vec{B}$ is the magnetic flux density. In this coupled MHD model, ${\vec{J}}_{L}$ is calculated with respect to the prescribed magnetic flux density.

(3) $${\vec{J}}_{L}=\sigma \times \vec{B}$$

The magnetic Reynolds number $R_{e_{m}}$, as shown in Equation (4), is integrated as a function of the induced and prescribed magnetic flux density.

(4) $$R_{e_{m}}=\mu_{0}\sigma UL$$

where $\mu_{0}$ is vacuum permeability, σ is electrical conductivity, U is flow velocity, and L is characteristic length.

### 2.3. Coupled Magneto-Hydrodynamics Model

The Lorentz force ${\vec{F}}_{L}$ is modeled with Equation (5) as a function of electric current density $\vec{J}$ and magnetic flux density $\vec{B}$. The Poisson equation for the electric potential φ is derived through the coupling of Olm’s law with the conservation of current as shown in Equation (6). Coupling the Poisson equation with the instantaneous velocity $\vec{v}$ and applied magnetic field ${\vec{B}}_{0}$ calculates electric current density $\vec{J}$, as shown in Equation (7). Electric current density is integrated into Equation (5) to solve for the Lorentz force, Equation (8), which is coupled with the conservation of momentum provided in Equation (2).

(5) $${\vec{F}}_{L}=\vec{J}\times \vec{B}$$

(6) $$\nabla^{2}\varphi =\nabla \cdot \left(\vec{v}\times {\vec{B}}_{0}\right)$$

(7) $$J=\sigma \;\left(-\varphi +\vec{v}\times {\vec{B}}_{0}\right)$$

(8) $${\vec{F}}_{L}=\sigma \;\left(-\nabla \varphi +\vec{v}\times {\vec{B}}_{0}\right)\times {\vec{B}}_{0}$$

The turbulence is modeled using the k-omega (k-ω) shear stress transport (SST) model, as shown in Equations (9) and (10).

(9) $$\frac{\partial }{\partial t}\left(\rho k\right)+\nabla \cdot \left(\rho k\bar{v}\right)=\nabla \cdot \left[\left(\mu +\sigma_{k}\mu_{t}\right)\nabla k\right]+P_{k}-\rho \beta f_{\beta^{*}}\left(\omega k-\omega_{0}k_{0}\right)$$

(10) $$\frac{\partial }{\partial t}\left(\rho \omega \right)+\nabla \cdot \left(\rho \omega \bar{v}\right)=\nabla \cdot \left[\left(\mu +\sigma_{\omega }\mu_{t}\right)\nabla \omega \right]+P_{\omega }-\rho \beta f_{\beta }\left(\omega^{2}-\omega_{0}{}^{2}\right)$$

The two k-ω equations are simultaneously evaluated to obtain the values of the turbulent kinetic energy k and the specific dissipation rate ρ, to model turbulent eddy viscosity $\mu_{t}$, where T is the turbulent time scale as shown in Equation (11).

(11) $$\mu_{t}=\rho kT$$

### 2.4. Boundary Condition and Geometry

**Figure 1:**
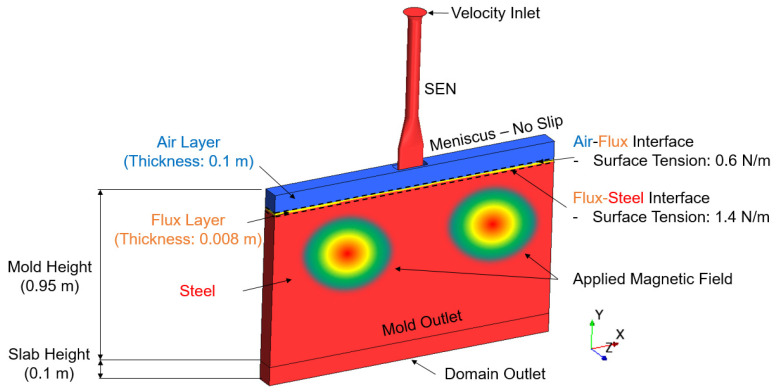
Computational domain and boundary conditions.

In order to make our model better predict the realistic flow field and the surface wave behavior in the primary cooling process, the geometry model was built based on the actual SEN and mold used by Nucor. The initial flux thickness and depth were also from the measured data of the Nucor caster. The boundary conditions utilized in the simulation are shown in Figure 1. Here, the velocity inlet, $u_{\mathrm{inlet}}$, is calculated utilizing the conservation of mass with respect to the density, as shown in Equation (12), where, $\rho_{tc}$ is the density at the torch cut off temperature, $\rho_{inlet}$ is the density taken at the temperature of the inlet, A is the cross section area of the mold, and ${\dot{V}}_{Steel}$ is the volumetric flow rate of steel. The surfaces of the narrow face, board face, and the mold outlet are set as walls. The prescribed magnetic flux density with respect to position was provided by industry collaborator, Nucor Corporation, and mapped to the mold.

(12) $$U_{\mathrm{inlet}}=\frac{\rho_{tc}}{\rho_{inlet}}A{\dot{V}}_{Steel}$$

For the computational study, the steel composition for the steel and flux layer is provided in Table 1. However, the material property of air was assumed based on the value extracted from the literature [14].

### 2.5. Electromagnetic Braking

**Figure 2:**
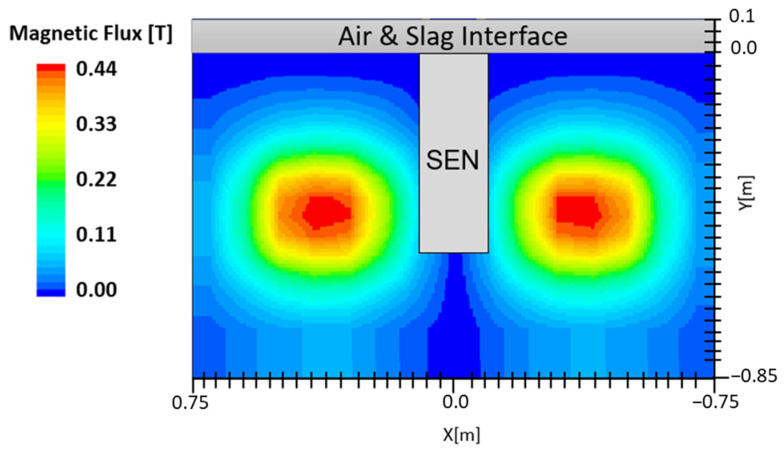
Applied magnetic field.

Electromagnetic forces generated by the EMBr unit can be applied as either static or moving magnetic fields in the mold. An example of a magnetic field applied in the mold is shown in Figure 2. The electromagnetic fields induced a current in the conducting molten steel, which generated Lorentz forces that oppose the flow and thus are referred to as “brakes” or “EMBr” [12,15]. The simulation utilizes a static EMBr configuration. In a static EMBr configuration, two circular magnetic fields are applied in the center of the mold normal to the broad face [8]. The purpose of this configuration is to dampen the molten steel as it recirculates around the applied EMBr. Electromagnetic forces are noted to offer an advantage over other flow control methods as the induced forces vary in strength, providing a theoretical self-stabilization for turbulent flow variations [3]. A gauss meter is utilized to measure the location and strength of the magnetic field. Per the MHD model, the magnetic field and location are defined in the STAR-CCM+ simulation conducted.

### 2.6. Computational Grid

**Figure 3:**
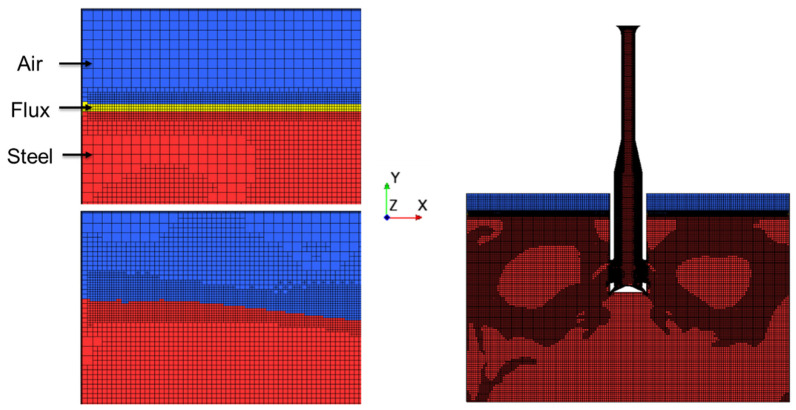
AMR simulation mesh.

Figure 3 shows the mesh utilized in the simulation. Utilizing adaptive mesh refinement (AMR), the optimal mesh size was selected for the simulation. The base mesh size was set as 5 mm, while the flux layer and nearby area were refined to 2.5 mm. By applying the AMR criteria, any cells with a velocity higher than 0.125 m/s were refined to 2.5 mm. This resulted in a grid with total of 15 million cells. This large computational grid with adaptive refinement ensured capturing a high flow gradient throughout the simulation. Per the computational iteration, AMR was defined to solve the governing equations while minimizing the computational grid without divergence. Further information on the theory and application of AMR can be found in the following literature by Shinji Sakane and Dinshaw Balsara [16,17].

### 2.7. Simulation Setup

**Figure 4:**
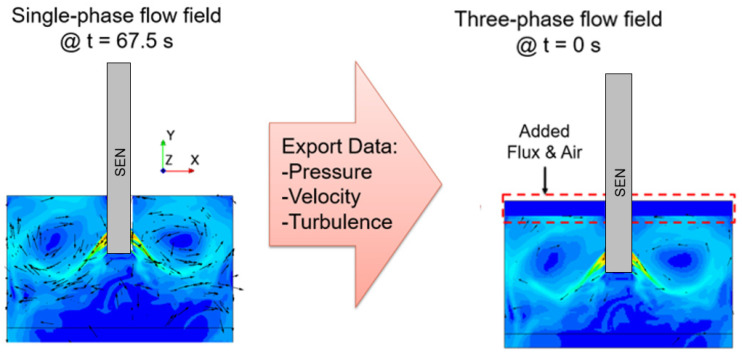
One-phase and three-phase simulation setup.

The simulations for the study were conducted in Simcenter STAR-CCM+ 2021.3.1, with a two-step approach, namely single-phase flow and three-phase flow. First, to reduce the computational time and increase the stability of the simulation, a single-phase flow simulation was conducted to develop the flow field. Next, as shown in Figure 4, a three-phase flow field was simulated with the pressure, velocity, and turbulence utilized as the initial conditions from the single-phase simulation output. For the single-phase simulation, the applied magnetic field and the molten steel material properties were defined. Once the flow field was developed, the output, as mentioned, was imported as the initial conditions for the three-phase simulation, incorporating the material properties of the flux and the air layers as well. The surface velocity and the surface wave height extracted from the simulation utilized time-averaged results, averaging the result over the computational time as mentioned in the literature by Liu et al. [18].

### 2.8. Design Optimization

To optimize the design of the mold according to the applied EMBr, the following study was conducted, as shown in Table 2. In order to ensure the fidelity and reliability of the simulation as much as possible, the parameters for SEN depth and mold width in this study were defined as the design parameters, which are realistic parameters used in the industrial practice of Nucor. The EMBr field was generated by a set of electromagnets, and the strength could be controlled by changing the current density of the electromagnet. A Gauss meter was used to measure the EMBr strength at different locations and a profile of the EMBr was generated and imported to the simulation. The parameters were integrated into the MHD model to analyze the impact on the surface wave. In the following study, the impact of SEN depth, mold width, and EMBr strength on the surface wave were investigated as they were believed to be the most important factors influencing the surface standing wave. The height of the mold throughout the simulation was kept constant at 0.95 m, as well as the material properties for each of the cases for air, slag, and steel layers.

## 3. Results and Discussion

### 3.1. Validation

**Figure 5:**
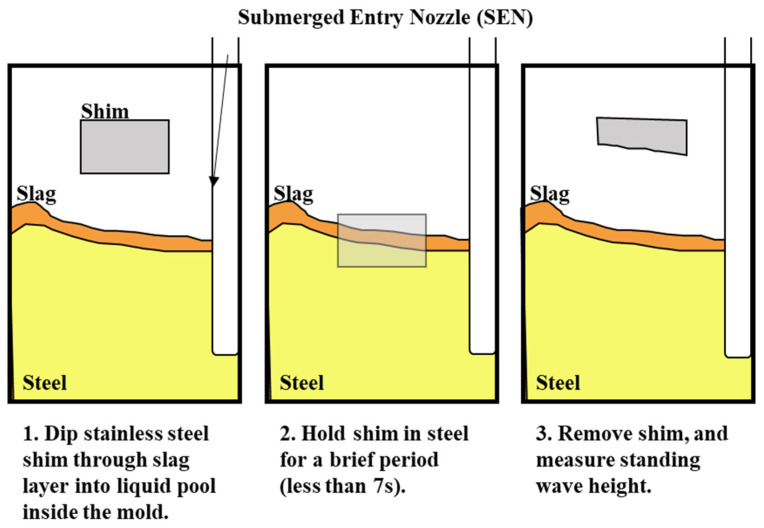
Shim dip test.

**Figure 6:**
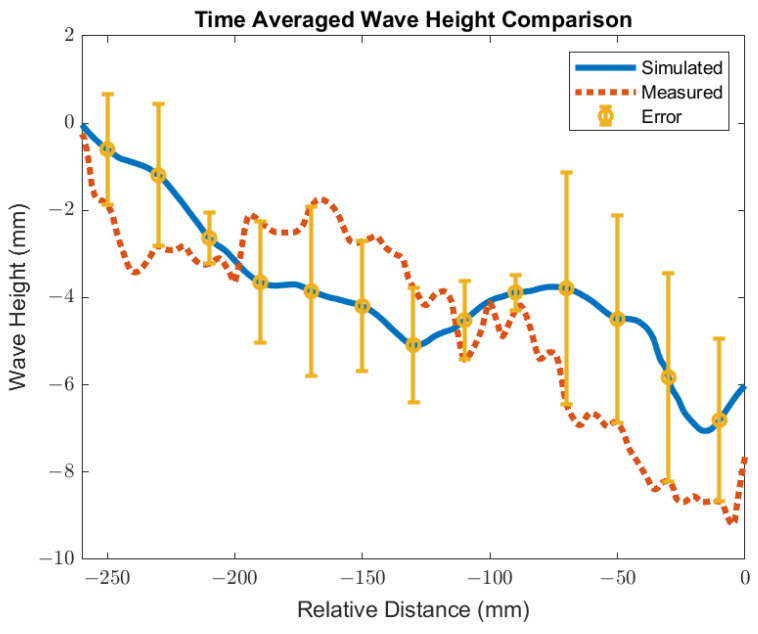
Time averaged wave height for validation.

The surface standing wave height generated from the baseline simulation was compared to the shim dip sample taken at the caster for validation. The purpose of the validation was to match the peak heights to validate the trend of the surface wave. The simulation was conducted with an EMBr center strength of 4450 G and casting speed of 3.43 m/min. Under the defined mold design and casting conditions, the height of the surface standing waves was captured and used for validation by measuring the oscillation marks due to the surface fluctuations in the shim dip test. Figure 5 shows the procedures of the shim dip test. A stainless steel shim was dipped in through the slag and into the steel. The portion of the shim that was immersed in the steel would melt. The shim that remained unmelted revealed the shape of the standing wave. The standing wave height was obtained by measuring the remainder of the shim sample. The same conditions were utilized in the simulation and the simulated wave height of the flux layer was compared with the shim dip measurements provided. As shown in Figure 6, the time averaged surface standing wave plotted against the measurements taken at the plant showed high confidence for the simulated results. Per the time averaged results from 10 s to 30 s, there was an average percentage difference of 16%, which showed a 1.58 mm average peak height difference. Per validation, the surface wave matched the trend provided.

### 3.2. Impact of Embr Strength

**Figure 7:**
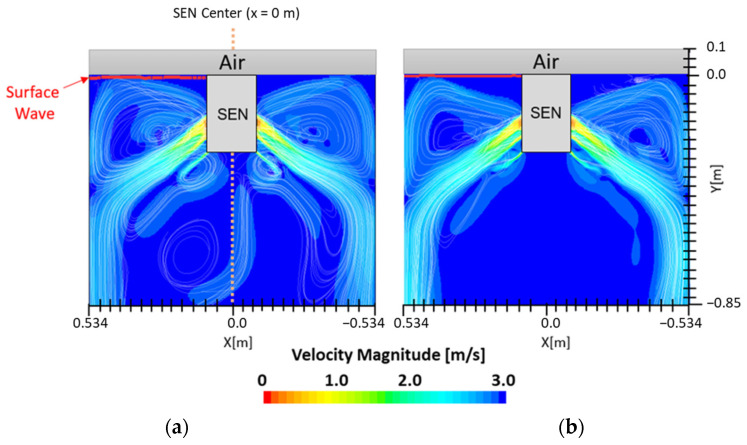
(**a**) Low EMBr strength 2975 G (Case 2) vs. (**b**) high EMBr strength 4350 G (Case 1) flow streamline.

Figure 7 shows the flow formation of Case 1 and Case 2. Here, the impact of EMBr on the surface wave was very evident. Case 1 utilized a stronger EMBr strength of 4350 G, while Case 2 utilized a lower EMBr of 2975 G. The height was kept constant at 0.95 m, the width was kept constant at 1.067 m, and the SEN depth was kept constant at 110 mm. In Case 1, because of the larger magnitude of the EMBr strength, the flow was recirculating around a larger magnetic field. Based on the figures, the impact of EMBr was clearly visible on the flow formation. In the mentioned plot, the horizontal distance measured started at the SEN center at x = 0 m to the mold wall in the left direction at x = 0.534 m. The recirculation zone of the molten steel was around the applied magnetic field. A larger EMBr resulted in the flow being directed more downwards and there was less impact on the surface wave.

**Figure 8:**
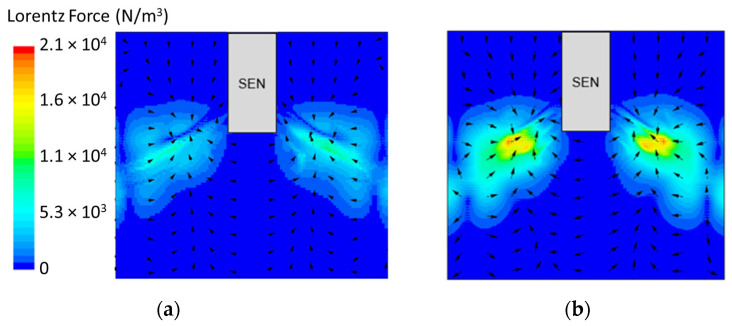
(**a**) Low EMBr strength 2975 G (Case 2) vs. (**b**) high EMBr strength 4350 G (Case 1) Lorentz force distribution.

**Figure 9:**
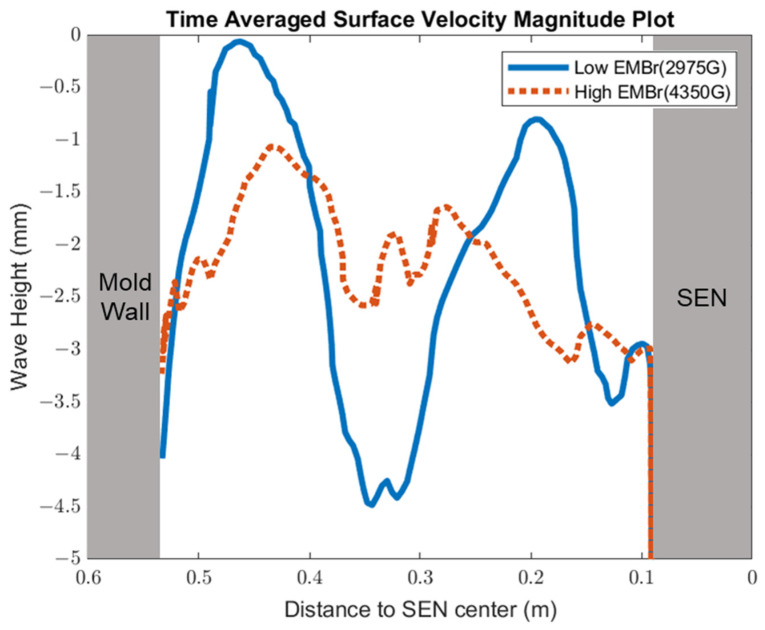
High EMBr strength (Case 1) vs. low EMBr strength (Case 2) standing wave comparison.

**Figure 10:**
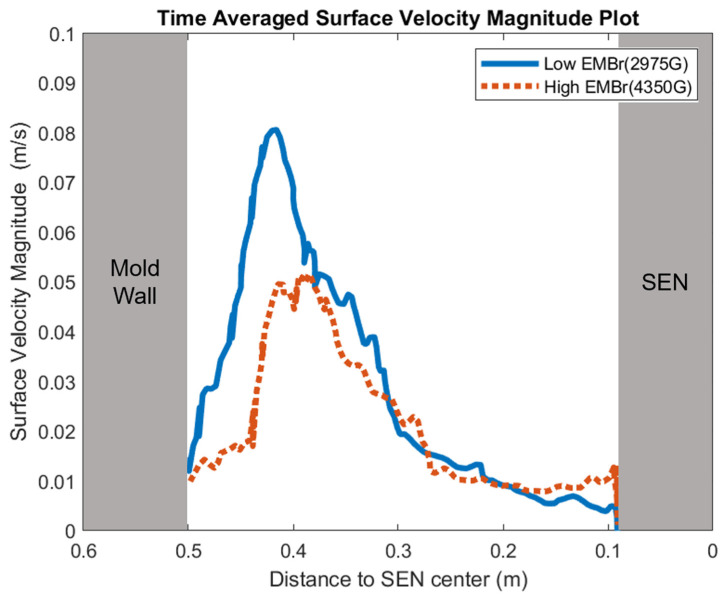
High EMBr strength (Case 1) vs. low EMBr strength (Case 2) standing velocity comparison.

Figure 8 shows the applied Lorentz force due to the EMBr. At a lower EMBr strength, there was less Lorentz force acting on the molten steel, thus the flow was more cluttered towards the upper mold. This ultimately led to a larger fluctuation of the surface standing wave in the lower EMBr case, as shown in Figure 9, because of the larger magnitude of surface velocity and lower Lorentz force acting against the molten steel, as shown in the plot in Figure 10. A larger EMBr strength dampens the flow, and the injected molten steel tends to flow downwards. This is evident when comparing the surface velocity. A larger EMBr strength reduced the average surface velocity magnitude by 15% relative to the lower EMBr strength. When comparing the surface standing wave of Case 1 and Case 2, Figure 9 shows the impact of EMBr strength. Case 2, which had a lower strength of EMBr, has a larger fluctuation of time averaged surface standing wave with a peak height of 4.8 mm and average wave height of 2.4 mm. While the higher strength of EMBr had a peak height of 3.01 mm and average surface standing wave of 2.1 mm. Increasing the EMBr strength from 2975 G to 4350 G decreased the average surface standing wave height by 12.5%.

**Figure 11:**
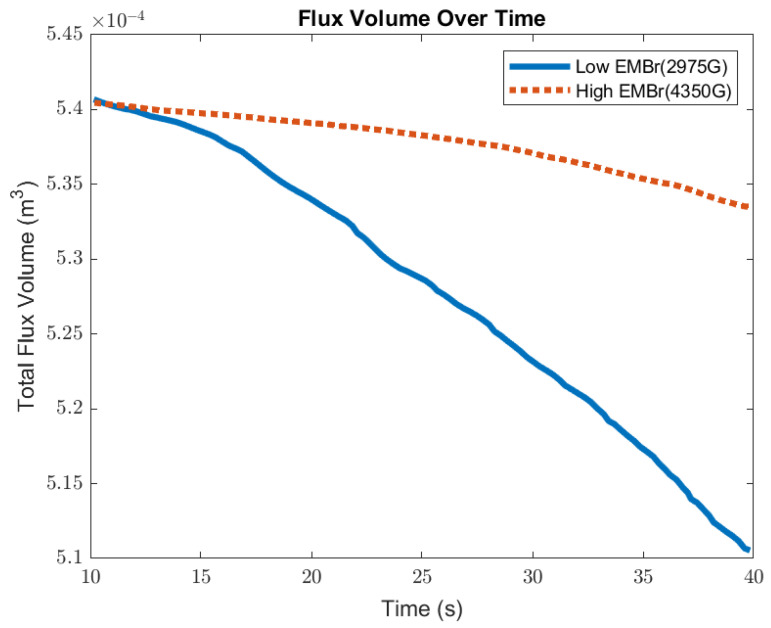
High EMBr strength (Case 1) vs. low EMBr strength (Case 2) flux volume comparison.

Figure 11 shows the comparison of the volume of flux between a low EMBr strength and high EMBr strength. With the defined surface tension, due to a larger surface wave, the flux layer sheared off and was carried away from the interface at the meniscus. The flux entrained into the steel degraded the steel quality. When comparing the impact of the surface standing wave on the flux layer, at a lower EMBr strength, the volume of the flux decreased by 8 × 10^−7^ m^3^ per second, while at the higher EMBr strength the flux layer decreased by 2 × 10^−7^ m^3^ per second. Increasing the EMBr strength reduced the flux entrainment by 4.25%. The differences obtained in the flux and wave parameters for Cases 1 and 2 are summarized in Table 3.

### 3.3. Impact of Submergence Depth

**Figure 12:**
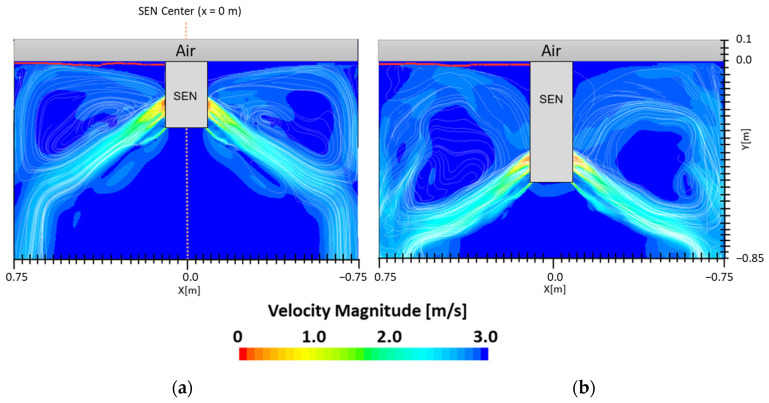
(**a**) Low SEN depth 110 mm (Case 4) vs. (**b**) high SEN depth 350 mm (Case 8) flow streamline.

**Figure 13:**
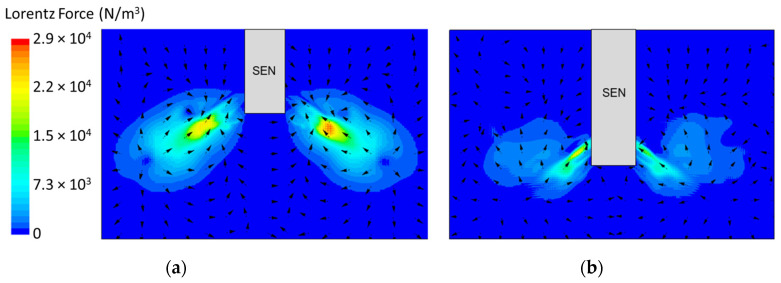
(**a**) Low SEN depth 110 mm (Case 4) vs. (**b**) high SEN depth 350 mm (Case 8) Lorentz force distribution.

In the following study, two different submergence depths were simulated to investigate the impact on flow. The first simulation, Case 4, utilized a submergence depth of 110 mm and was noted as having a lower SEN depth. The second simulation, Case 8, utilized a nearly three times larger submergence depth of 350 mm and was noted as having a higher SEN depth. Both were at a constant EMBr strength of 2975 G. Figure 12 shows the flow formation and the corresponding velocity magnitude at the surface of the lower and higher SEN depth. Increasing the submergence depth with a constant magnitude of EMBr strength applied resulted in the jet penetrating deeper into the mold. At a higher SEN, the molten steel injection was below the applied EMBr. Therefore, there was less Lorentz force acting against the melt to dampen it, as shown in Figure 13.

**Figure 14:**
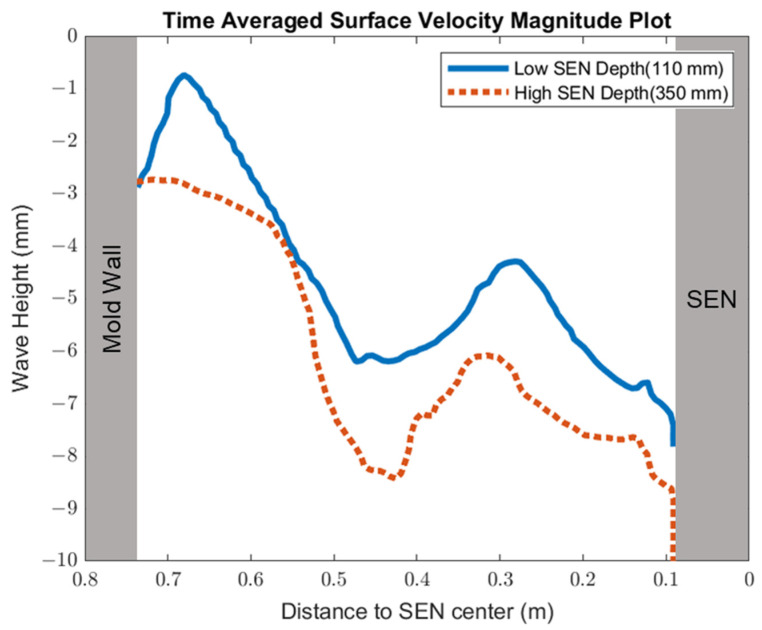
Low SEN depth (Case 4) vs. high SEN depth (Case 8) standing wave comparison.

**Figure 15:**
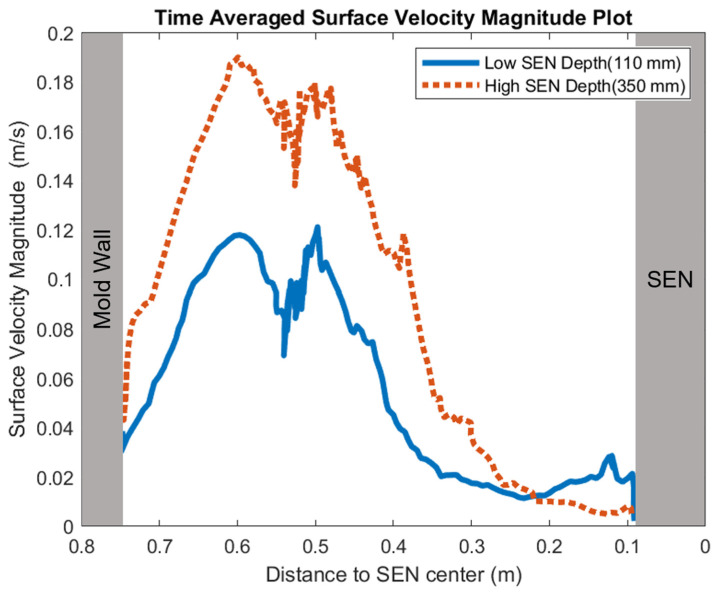
Low SEN depth (Case 4) vs. high SEN depth (Case 8) surface velocity comparison.

**Figure 16:**
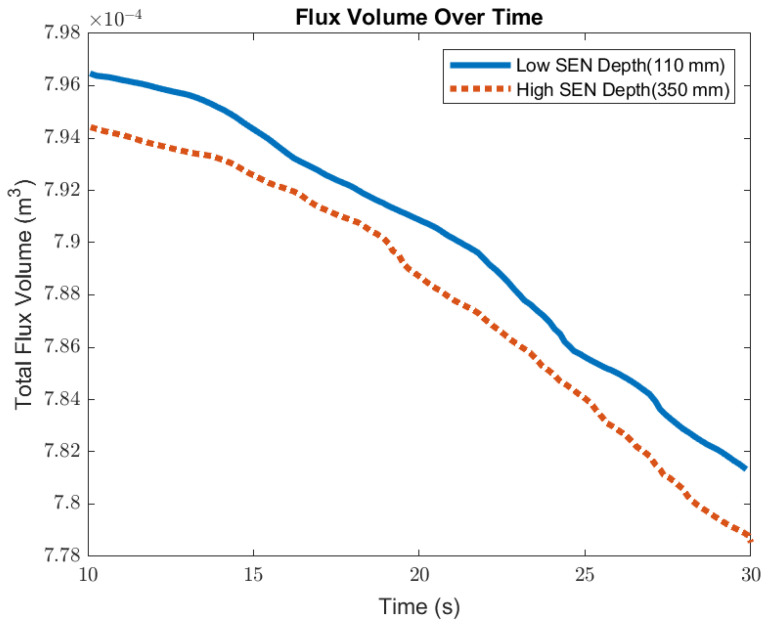
Low SEN depth (Case 4) vs. high SEN depth (Case 4) flux volume comparison.

Figure 14 shows the surface standing wave height comparison. A higher SEN depth increased the height of the recirculation zone, which was more cluttered with more molten steel. This resulted in a larger amplitude for the surface standing wave. With a lower SEN depth, the flow was directly impacted by the Lorentz force dampening it. As shown in Figure 15, as the flow recirculated, the surface velocity was larger for the high SEN depth relative to the low SEN depth. With the average velocity magnitude at the surface increasing by 30% with the increase in SEN depth, as mentioned, at a lower SEN, the EMBr was applied directly next to the injection port of the SEN. The peak surface standing wave for a low SEN depth was 7.04 mm, with an average height of 4.29 mm. While for a high surface standing wave the peak was 8.55 mm, and the average surface standing wave height was 5.12 mm. When the SEN depth was increased from 110 mm to 350 mm, the average height increased by 19%. Figure 16 shows the total flux volume in the mold comparison between a low and high SEN depth. At 110 mm, the flux volume decreased by 6 × 10^−7^ m^3^ per second while at 350 mm, the flux volume decreased by 8 × 10^−7^ m^3^ per second. Increasing the SEN depth increased the flux entrainment by 2.6% (Table 4).

### 3.4. Impact of Mold Width

**Figure 17:**
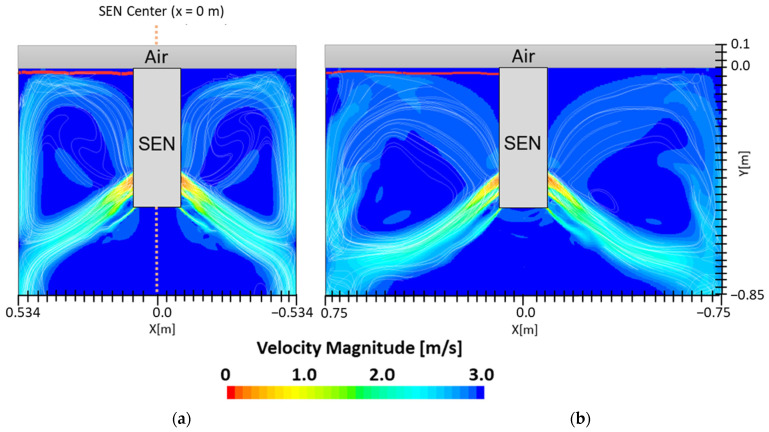
(**a**) Low mold width 1.067 m (Case 5) vs. (**b**) high mold width 1.50 m (Case 7) flow streamline.

**Figure 18:**
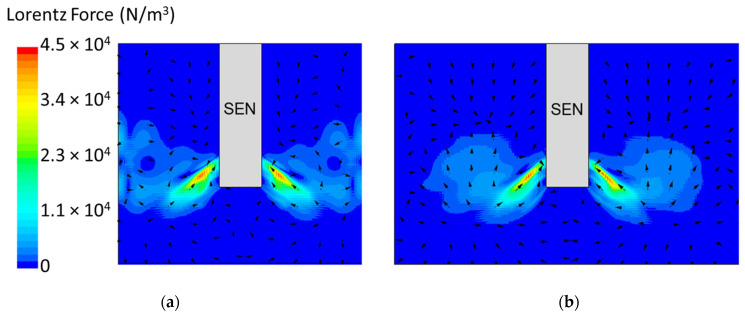
(**a**) Low mold width 1.067 m (Case 5) vs. (**b**) high mold width 1.50 m (Case 7) Lorentz force distribution.

**Figure 19:**
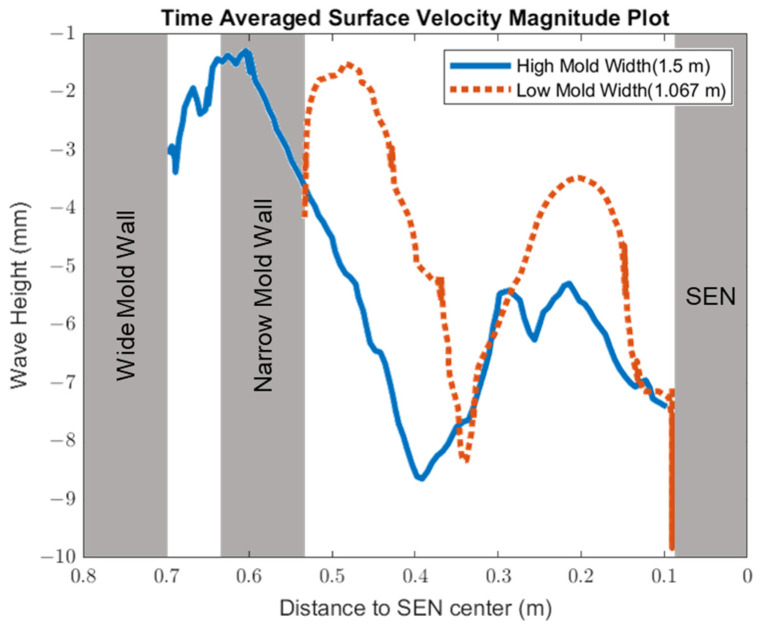
Low mold width (Case 5) vs. high mold width (Case 7) standing wave comparison.

**Figure 20:**
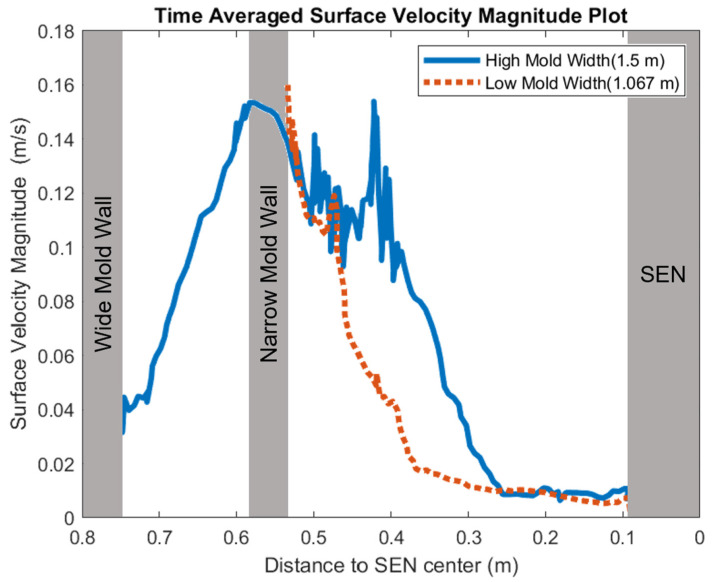
Low mold width (Case 5) vs. high mold width (Case 7) surface velocity comparison.

**Figure 21:**
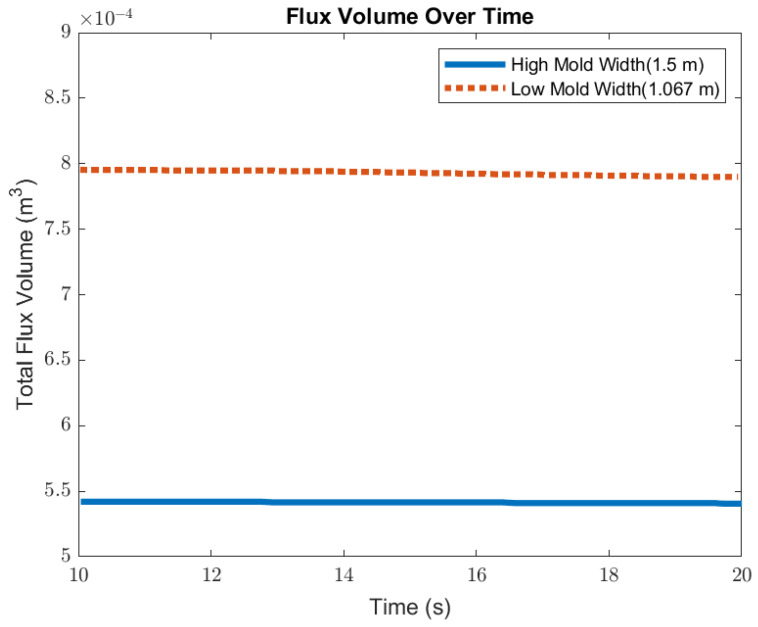
Low mold width (Case 5) vs. high mold width (Case 7) flux volume comparison.

In this section, mold widths of 1.067 m and 1.50 m were utilized as the geometry domain. Reducing the mold width lowered the surface area for the molten steel to recirculate, as shown in Figure 17. The flow within each mold design was simulated at a constant EMBr strength and SEN depth. Figure 17 provides the flow formation for the different mold widths, while Figure 18 shows the applied Lorentz force. Based on the figure, Case 5 with the lower mold width has a larger surface area, where the Lorentz force is applied to dampen the flow, compared with Case 7 with a larger mold width. With all of the constant parameters, per the applied EMBr, reducing the mold width lowered the surface velocity due to the larger surface area coverage of the overall molten steel injected. As shown in Figure 19, at a lower mold width (Case 5), the peak surface standing wave height was 8.37 mm with an average wave height of 4.48 mm, while at the higher mold (Case 8) width, the peak surface standing height was 8.65 mm, and the average height was 4.87 mm. This was due to the larger surface velocity per the Lorentz force induced. As shown in Figure 20, the average surface velocity increased by 22% when increasing the mold width from 1.067 m to 1.50 m. Increasing the mold width increased the average surface standing wave height by 8.71%. Figure 21 shows that, at a higher mold width, the flux volume decreased by 8 × 10^−7^ m^3^ per second while at a lower width the flux decreased by 8 × 10^−8^ m^3^ per second. Reducing the mold width increased the slag entrainment by 0.9% (Table 5).

## 4. Conclusions

In industrial practice, in order to prevent excessive surface wave causing surface defects, EMBr is applied to generate a Lorentz force that dampens the molten steel. In this research, utilizing the developed MHD model, application of EMBr was simulated based on a realistic Nucor caster. The model was validated to accurately predict the behavior of the surface standing wave under certain casting conditions. By using the two-step approach, including single-phase and three-phase simulations, the stability of the simulation was ensured and the computational time was reduced. There are three major parameters, EMBr strength, SEN submergence depth, and mold width, that can be optimized by a manufacturer to optimize the steel quality by reducing the surface wave. They were analyzed for their impact on the surface standing wave. The validation case was simulated with 1.58 mm average difference from the measured plant data. It was found that increasing the EMBr strength from 2975 G to 4350 G decreased the average surface standing wave height by 12.5%. At a lower EMBr strength, the flux volume decreased by 8 × 10^−7^ m^3^ per second, while at the higher EMBr strength, the flux layer decreased by 2 × 10^−7^ m^3^ per second. Increasing the EMBr strength reduced the flux entrainment by 4.25%. Increasing the submergence depth increased the height of the recirculation zone around the center of the applied EMBr. When the submergence depth was increased from 110 mm to 350 mm, the average height increased by 19% and the flux entrainment increased by 2.6%. Increasing the mold width from 1.067 m to 1.50 m increased the average surface standing wave height by 8.71% and the slag entrainment by 0.9%. Reducing the surface defects is feasible by applying the appropriate conditions. It is also evident that the application of EMBr is a critical method to regulate molten steel. Increasing the EMBr strength had the largest impact on reducing the surface standing wave, then decreasing the submergence depth, and lastly reducing the mold width, which had the lowest impact. This article provides a practical approach to optimize EMBr-related settings. Although this paper only considered the direct impacts of the three key parameters, future study could look at ideal EMBr strength per defined condition, replacing physical work with numerical simulations to improve steel quality.

## Figures and Tables

**Table 1:** Material properties.

Material Properties	Steel	Air	Flux
Viscosity (Pa-s)	0.03	1.855 × 10^−5^	0.09
Density (kg/m^3^)	7003.016	1.184	2767.99

**Table 2:** Case matrix.

Case	SEN Depth (mm)	Width (m)	Casting Speed (m/min)	EMBr (G)
1	110	1.067	3.18	4350
2	110	1.067	3.18	2975
3	110	1.50	3.18	4350
4	110	1.50	3.18	2975
5	350	1.067	3.18	4350
6	350	1.067	3.18	2975
7	350	1.50	3.18	4350
8	350	1.50	3.18	2975

**Table 3:** Comparison of cases with high and low EMBr, namely Cases 1 and 2.

Results	Low EMBr 2975 G Case 2	High EMBr 4350 G Case 1	Diff.
Peak Wave Height	4.8 mm	3.01 mm	59.47%
Average Wave Height	2.4 mm	2.1 mm	12.50%
Volume of Flux Rate of Decrease	8 × 10^−7^ m^3^ per s	2 × 10^−7^ m^3^ per s	4.25%

**Table 4:** Comparison of cases with a high and low SEN depth, namely Cases 4 and 8.

Results	Low SEN Depth 110 mm Case 4	High SEN Depth 350 mm Case 8	Diff.
Peak Wave Height	7.04 mm	8.55 mm	21.45%
Average Wave Height	4.29 mm	5.12 mm	19.00%
Volume of Flux Rate of Decrease	6 × 10^−7^ m^3^ per s	8 × 10^−7^ m^3^ per s	2.60%

**Table 5:** Comparison of cases with a high and low mold width, namely Cases 5 and 7.

Results	Low Width 1.067 m Case 5	High Width 1.50 m Case 7	Diff.
Peak Wave Height	8.37 mm	8.65 mm	3.35%
Average Wave Height	4.48 mm	4.87 mm	8.71%
Volume of Flux Rate of Decrease	8 × 10^−7^ m^3^ per s	8 × 10^−8^ m^3^ per s	0.90%

## Data Availability

Restrictions apply to the availability of the data presented in this study. Any further detail is subject to confidentiality for Nucor Steel Decatur, LLC.
